# A Stable and Dependable Visual Technique for On-Site Nipah Virus Nucleic Acids Detection

**DOI:** 10.1038/s41598-025-91593-w

**Published:** 2025-02-27

**Authors:** Wencong Chen, Li Cai, Danni Ye, Jiahao Chen, Xueyan Ai, Xuehua Tang, Anqi Deng, Zihan Gao, Meihua Xiang, Mingen Yu, Kun Zhu, Maopeng Wang

**Affiliations:** 1 Wenzhou Key Laboratory for Virology and Immunology, Institute of Virology, Wenzhou University, Chashan University Town, Wenzhou, 325000 China; 2 Institute of Animal Science of Zhuji, Zhuji, 311800 China; 3 Research Unit of Key Technologies for Prevention and Control of Virus Zoonoses, Changchun Institute of Veterinary Medicine, Chinese Academy of Medical Sciences, Chinese Academy of Agricultural Sciences, Changchun, 130122 China; 4 Research and Development Department, Hangzhou Goodhere Biotechnology Co., Ltd, Hangzhou, 311100 China; 5 Beijing Origingene-tech Biotechnology Co., Ltd, 3rd Floor, Building of 4th,1 Area, North of Liandong U Zone, Beijing, 101102 China

**Keywords:** Nipah virus, On-site, One-step RT-PCR, Lateral-flow immunochromatography, Dual-target detection, Biological techniques, Molecular biology, Zoology

## Abstract

The Nipah Virus (NiV) is a zoonotic pathogen with the mortality rate of up to 75%, recurring in Asia over the past two decades. Due to increasing the risk of human transmission mediated by various intermediate hosts such as pigs and bats, it is necessary to produce an accurate and reliable point-of-care molecular detection method for NiV field diagnosis. In this study, we designed two pairs of primers targeting the conserved G and P genes and developed a point-of-care nucleic acid detection (POC-NAD) system by integrating one-step RT-PCR, lateral flow immunoassay, and microfluidic technologies. The POC-NAD system shows high specificity and sensitivity, with a Limit of Detection (LoD) of 199.1 copies/rxn. The primers aiming to the conserved sequences enables simultaneous detection of both NiV-M and NiV-B strains. Continuous evaluation of 21 simulated clinical samples demonstrated 100% concordance with RT-PCR results. Lateral flow-based visualization improves the display time and legibility of RT-PCR results. Additionally, microfluidic chips or chambers offer disposable reagent containers and consistent PCR amplification results across various field conditions. Therefore, the diagnostic tool is suitable for real-time nucleic acid testing and NiV surveillance in resource-limited field environments.

**Supplementary Information:**

The online version contains supplementary material available at 10.1038/s41598-025-91593-w.

## Background

Nipah virus (NiV) was first known in 1998 in Malaysia and was later reported by Singapore, where the transmission was suspected to occur through direct contact with sick pigs or their contaminated tissues^1,^^2^. In Malaysia, more than 1 million pigs were sacrificed to keep control of the outbreak but even 105 out of 265 people died, which caused serious economic damage^3,^^4^. The Nipah virus is a fatal RNA virus classified as a biosafety level 4 pathogen. It transmits between humans and animals to cause neurological and respiratory diseases^5^. Two presentative viral strains of NiV, namely NiV-B and NiV-M, were identified separately from some isolates in Bangladesh, India, and Malaysia. Although they share 91.8% homology similarities, the NiV-B strain is verified to have a higher fatality rate^6,^^7^ with ranging from 40 to 75%.

The present detection methodologies for the Nipah virus include antibody-based and nucleic acid-based molecular diagnostic technology. The former one includes enzyme-linked immunosorbent assay (ELISA) and neutralization test^8^. The viral N protein is usually used as a unique target, but rapid viral genome mutations and antibody affinity may compromise its sensitivity and accuracy. Although neutralization tests can detect Nipah virus antibody neutralizing activity, the requirement for BSL-4 laboratory facilities limits their clinical testing application^9^. Polymerase chain reaction (PCR) technology is primarily used for its high sensitivity, specificity, and rapid detection in the later one. RT-PCR and TaqMan RT-PCR targeting the Nipah Virus N gene are previously established and commonly used for diagnosis^10,^^11^. These tests can rapidly and accurately quantify the Nipah virus in samples. As a highly expressed viral gene, the N gene serves as a target for nucleic acid detection, which can enhance assay sensitivity^12,^^13^. However, assays targeting the N gene cannot distinguish between viral genomes and mRNA transcripts in both in vitro and in vivo samples, hindering precise viral genome quantification. Jensen established the RT-qPCR assay to solve this problem by targeting the non-coding region genes between the F and G genes of the Nipah virus^14^. It proves that novel amplification target for Nipah virus is still needed to monitor the viral transmission risk. Besides, the NiV transmission via pig movement networks is not negligible, further expanding the viral infection field or scenario^15^. Current detection methods, reliant on costly equipment, are challenging to implement in areas prone to Nipah virus outbreaks, which often have inadequate sanitation and limited resources. Therefore, developing a Point of Care Technology (POCT) method to detect the Nipah virus in pigs and other wild animals is crucial^16^.

In this study, we compared all the Nipah virus genome sequences from the NCBI dataset. Through evolutionary tree analysis, two genotypes were classified. Numerous conserved sequences were found in the structural protein-encoding genes such as P and G gene, and viewed as potential universal primers design target for both Nipah genotypes. Consequently, we targeted the G and P genes to develop a novel, colorimetric dual-target detection strategy for monitoring replicable Nipah virus particles, by utilizing one-step RT-PCR in conjunction with lateral-flow strips and affordable, portable PCR devices.

## Materials and methods

### DNA/RNA extraction

Viral nucleic acid is extracted by DNA/RNA co-extraction kit-magnetic bead method (Niu-gene, M063-100) according to the instructions. Briefly, the sample is first mixed with protease K and lysate (VLr), followed by incubation with magnetic beads for 5 min. Then, magnetic rock was used to absorb magnetic beads and capture DNA/RNA. Finally, the DNA/RNA was dried for 1 min, eluted with buffer and transferred into a new EP tube.

### Portable PCR instrument and one step RT-PCR

**Fig. 1:**
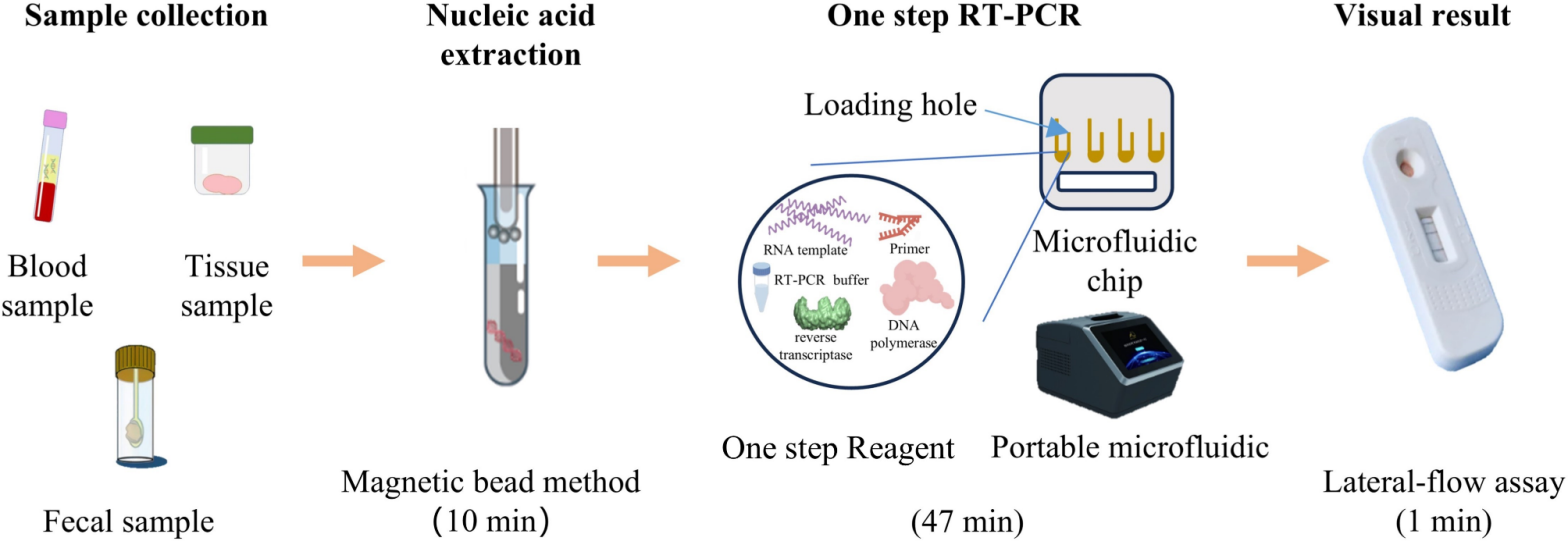
The process of POC-NAD from nucleic acid extraction to result reading.

The portable PCR system utilizes a microfluidic chip (provided by Beijing Origin Biotech) to complete amplification, distinguishing it from conventional PCR systems. This integrated design enables reliable amplification even under suboptimal field conditions. The one-step RT-PCR refers to the direct reverse transcription of viral RNA into cDNA and amplifies in the presence of reverse transcriptase, DNA polymerase, UDG enzyme, RNase inhibitor enzyme, reaction buffer (including Mg2+, dUTP, primers, etc.) and the template, without the need for a two-step reaction. This unified approach eliminates RNA handling between reactions thereby significantly reducing contamination risks and false-positive outcomes.All enzymes and reagents were sourced from Fapon Biotech. The complete thermal cycling protocol requires 47 min (Fig. 1).

### Viral genomes

Viral genomes used for a one-step PCR assay were stored in the laboratory, including African swine fever virus (ASFV), Porcine reproductive and respiratory syndrome virus (PRRSV), Porcine epidemic diarrhea virus (PEDV), porcine rotavirus (PRoV), Porcine foot-and-mouth disease virus (OZK93), Porcine delta coronavirus (PDCoV), and Transmissible gastroenteritis virus (TGEV).

### RNA reference Preparation

The P and G gene fragments were synthesized by Sangon Biotech and transcribed in vitro by using the T7 High Yield RNA Transcription Kit (Vazyme) and following the manufacturer’s protocol. RNA was purified by using the Trizol method, and the RNA concentration was determined by using the NanoDrop 2000 spectrophotometer (Thermo Fisher).

### Genotyping analysis and primer design

Sixty-four NiV complete sequences in different species’ reservoirs from India, Malaysia, Bangladesh, Thailand, Cambodia, and Indonesia were selected in NCBI(National Center for Biotechnology Information (nih.gov)) and sequences aligned by using MAFFT(MAFFT - a multiple sequence alignment program (cbrc.jp)) and MEGA 7.0. Tree file results were analyzed by using MEGA 7.0 and iTOL (iTOL: Interactive Tree Of Life (embl.de)). According to the conserved sequences of the NiV G and P genes, primer sequences were designed (Table 1). All the sequences’ list is seen in the Supplementary 1.

**Table 1:** Primer sequences.

Primer name		Primer sequence 5’−3’
P-1 F:		5’-dig-TGTCGAACACCCGTGACTG
P-1 R:		5’6’-FAM-ATCACTCCACCCTCTCTCAGG
P-2 F:		5’6’-FAM- AACCCACTCCCTTTTAGAGA
P-2 R:		5’-dig-TCACCTCTGTCTAGTACCTCTCC

### Point-of-care nucleic acid detection (POC-NAD) system

For POC-NAD, the volume of forward and reverse primers (10 mmol/µL, Sangon Biotech) used for the amplification reaction is 0.2 µL. The respective volumes of the template, enzyme mix, and buffer mix are 5 µL, 0.6 µL, and 14 µL. The total volume for the system is 20 µL. Reaction conditions include reverse transcription at 55 ℃ for 5 min, enzyme inactivation at 95 ℃ for 2 min and 30 s, followed by thermal activation of Taq enzyme and predenaturation, with 35 cycles (95 ℃ for 5 s, 60 ℃ for 20 s).

### Lateral flow immunochromatography

**Fig. 2:**
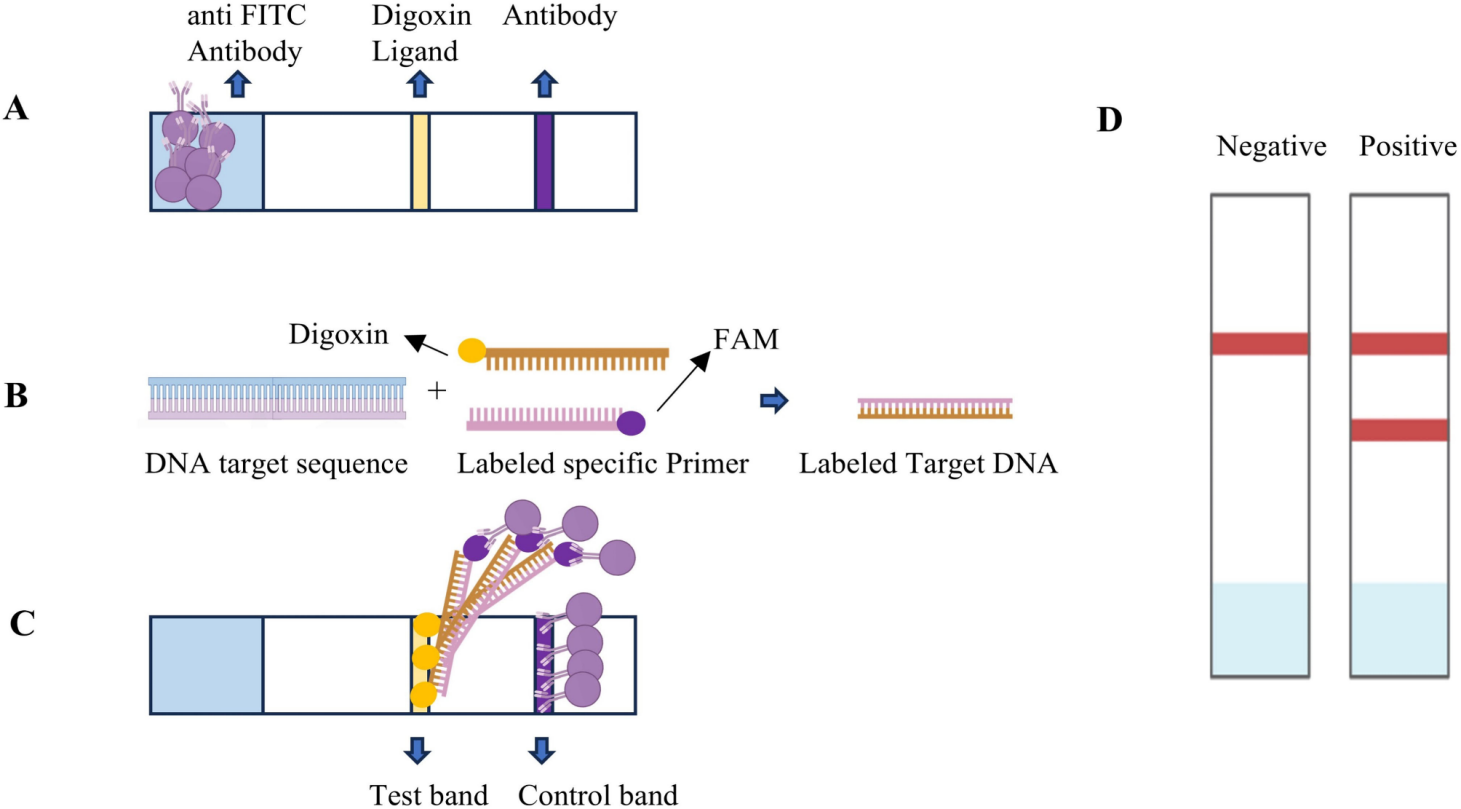
The principle of double antigen sandwich colloidal gold immunochromatography. (**A**) Colloidal gold-anti-FAM antibody (mouse anti-FAM) was placed on the binding pad. The test (T) line of anti-Digoxin antibody was fixed as the detection line. A fixed sheep anti-mouse polyclonal antibody served as (control) C line, which can combine mouse antibody; (**B**) In the presence of the target sequence, the amplified product, marked with Digoxin and FAM, can be generated using specific primers; (**C**) In the presence of the target product, a “colloidal gold - anti-FAM antibody - target product - anti-Digoxin antibody structure” was formed at the T-line to make the T-line color. At the position of line C, “Colloidal gold - anti-FAM antibody - sheep anti-mouse polyclonal antibody” was formed to make line C color; (**D**) Negative and positive results.

The results were visualized by using the colloidal gold double antigen sandwich methodology^17^, which fixed specific antibodies at the binding pad, test line, and control line, and marked the corresponding lable at the 5’ end of the primer (Fig. 2). The test paper is provided by goodhere biotech Co.,Ltd.

### Specificity of POC-NAD

The specificity of POC-NAD was assessed, and the DNA genome (ASFV), as well as the RNA genomes (PEDV, PRRSV, PDCoV, RoV, TGEV, and OZK93) as sample templates, were used in a one-step RT-PCR reaction under optimized conditions. The RNA fragments of G and P genes were performed as the positive control and RNase-free water (Vazyme) was used as the negative control. We repeated specificity test three times.

### Sensitivity of POC-NAD

NiV-G and NiV-P RNA templates transcribed in vitro were continuously diluted 2-fold with using nuclease-free water, and sensitivity was measured from 1592.5 copies/rxn to 99.55 copies/rxn. We repeated sensitivity test three times. The limit of detection (LoD) was determined which was based on 20 replicates with using a 2× diluted RNA template. And nuclease-free water was used as a no-template control.

The control line was developed to confirm the validity of the assay, with all test strips imaged within 1 min using a standardized protocol. Quantitative analysis via ImageJ software demonstrated a statistically significant positive correlation between the optical density of colloidal gold test lines and viral nucleic acid copy numbers.

**Concentration and copy number conversion formula**:

$$\:y(copies/\mu\:L)\:=\:\left[x\right(ng/\mu\:L)/(base\:number\times\:340) ]\times\:6.02\times\:{10}^{14}$$

### Detection of simulated clinical sample

To assess the accuracy of the established method for analyzing clinical samples, it was compared with the RT-PCR method. Common Intestinal tissue samples were mixed with the target RNA fragment (2.5µL for clinical sample RNA and 1.25µL for each target RNA fragment in 6370 copies/µL) of the Nipah virus. The sample, which was diluted 1:1 with ddH_2_O, was used as the negative simulated clinical sample. While the former served as the positive simulated clinical sample. The positive and negative controls were G and P genes RNA and H_2_O, respectively. The results were observed by using 1% agar-gel electrophoresis and lateral flow strips. Kappa consistency analysis was performed for RT-PCR and POC-NAD^18^.

**Kappa formula (**PA represents the probability of observing agreement and PE represents the probability of expecting agreement**)**:

$$\:\varvec{K}\varvec{a}\varvec{p}\varvec{p}\varvec{a}=\frac{\varvec{P}\varvec{A}-\varvec{P}\varvec{E}}{1-\varvec{P}\varvec{E}}\varvec{}$$

## Result

### Genotyping analysis and primer design

**Fig. 3:**
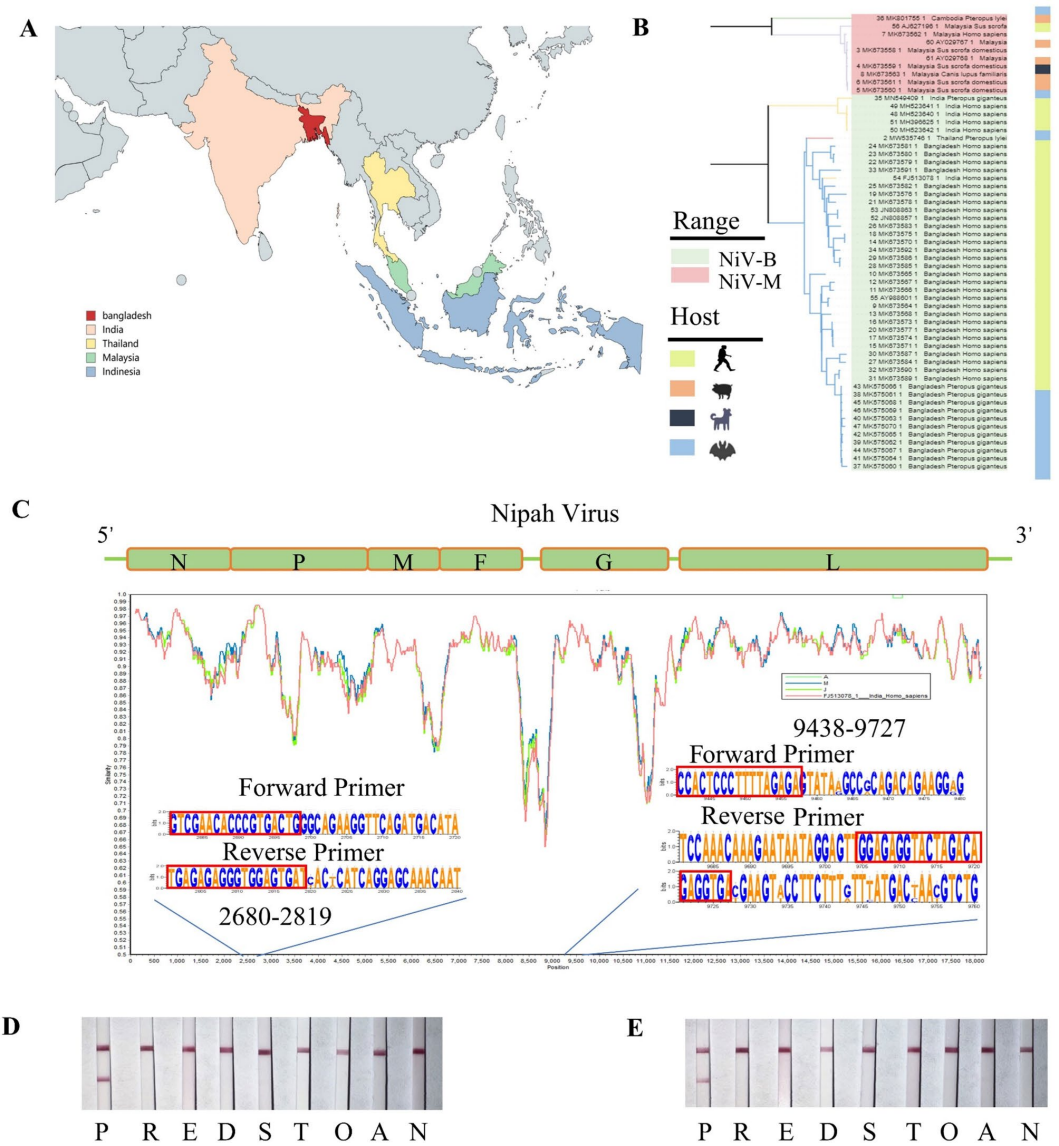
Comparison of Nipah virus genome and validation of specificity of POC-NAD. (**A**) Epidemiological region of Nipah virus; (**B**) A phylogenetic tree based on sixty-four complete genomic sequences of the Nipah virus; (**C**) The similarity of sixty-four complete genomic sequences was shown with WebLogo and demonstrated by using Simplot v 3.5.1 with NC002728 as the query sequence; (**D**) Results of the specificity of P-1 on lateral flow assay; (**E**) Results of the specificity of P-2 on lateral flow assay; P: Positive RNA template (3185 copies/rxn); R: PRoV; E: PEDV; D: PDCoV; S: PRRSV; T: TGEV; O: OZK93; A: ASFV; N: Negative control. The experiment was repeated three times.

To find potential target sequences, we compared sixty-four complete Nipah virus genome sequences from NCBI, including both NiV-B and NiV-M genotypes which were currently reported from India, Malaysia, Bangladesh, Thailand, Cambodia, and Indonesia (Fig. 3A). In this study, the P and G genes were selected as detective targets (2681 bp to 2801 bp and 9438 bp to 9727 bp) and the design of the two pairs of primers are highly conserved. Therefore, it is theoretically effective to detect both genotypes of Nipah (Fig. 3B and C).

### Specificity of POC-NAD

In this section, we conduct a specific test for the POC-NAD to be established. Positive controls showed results on lines T and C, while the negative control group and other virus template groups displayed negative results, banding was found only on line C (Fig. 3D and E). Two other sets of experiments repeated the same results (Supplement 2).

### Sensitivity of POC-NAD

**Fig. 4:**
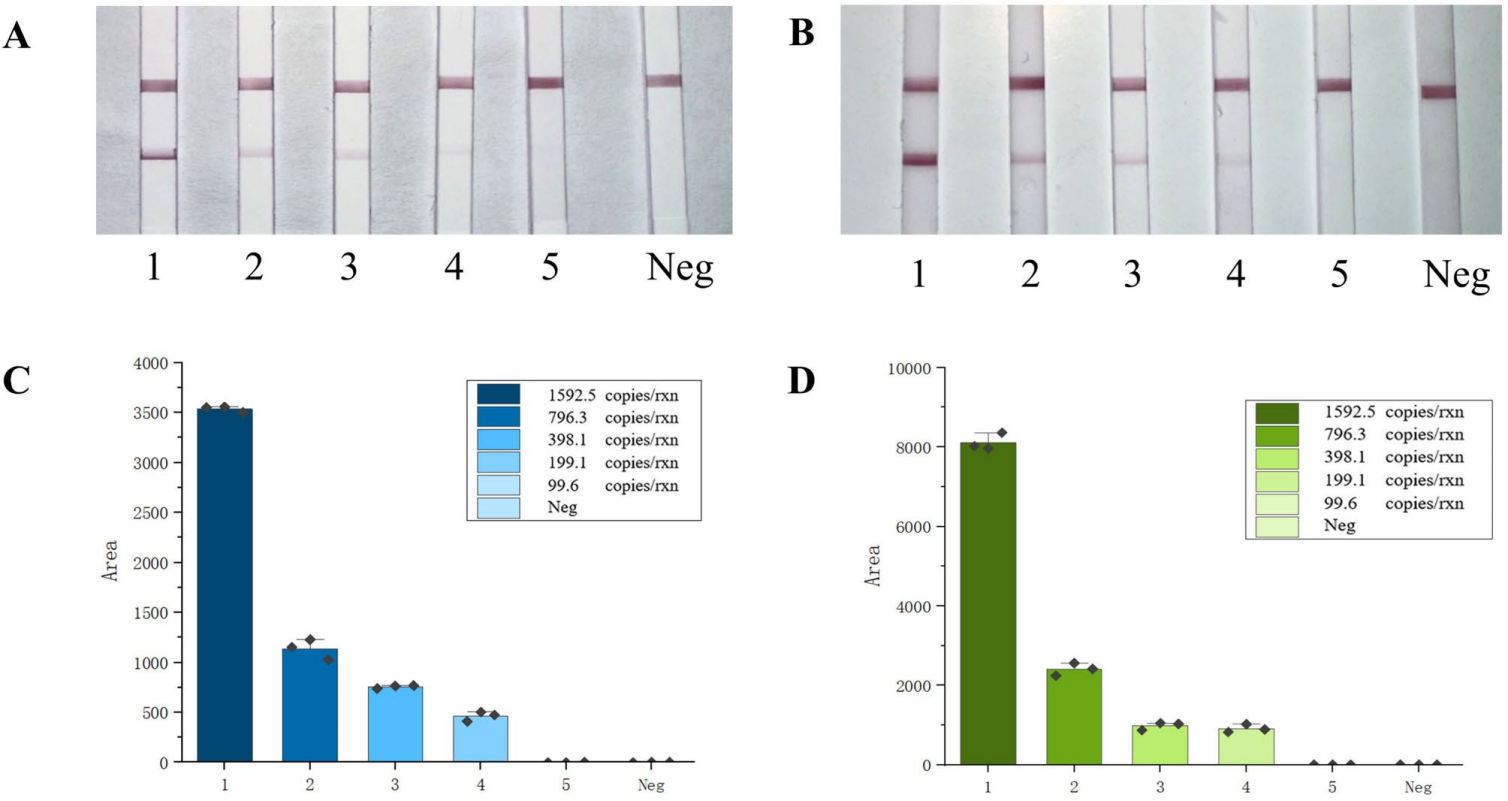
Sensitivity validation of POC-NAD. (**A**) The visual result of P-1; (**B**) The visual result of P-2; C, D. The visual outcomes of various concentrations were subjected to grayscale analysis, an initial quantitative analysis can be conducted by using this method. 1–5: 1592.5 copies/rxn, 796.25 copies/rxn, 398.1 copies/rxn, 199.1 copies/rxn, 99.55 copies/rxn; Neg represents the negative control. The experiment was repeated three times (supplement 2).

To verify the sensitivity of POC-NAD to NiV detection, RNA fragments of NiV-G and NiV-P transcribed in vitro ranging from 1592.5 copies/rxn to 99.55 copies/rxn were used as templates. The results showed that both P-1-X and P-2-X displayed positive results on the lateral flow strip at the positive template concentrations above 199.1 copies/rxn. When the template concentrations are below 99.55 copies/rxn, the two primers failed to show positive results in the POC-NAD (Fig. 4A and B). From the gray analysis, we visualized the detection limit and provided a potential quantitative method based on bands intensity of different concentrations (Fig. 4C and D).

LoD for NiV was determined by identifying the minimum concentration of NiV RNA template, which could be detected in over 95% of the 20 replicates (Supplement 2). According to the results, all replicates were tested to be positive when the template concentration is 199.1 copies/rxn.

### Detection of simulated clinical specimens

**Fig. 5:**
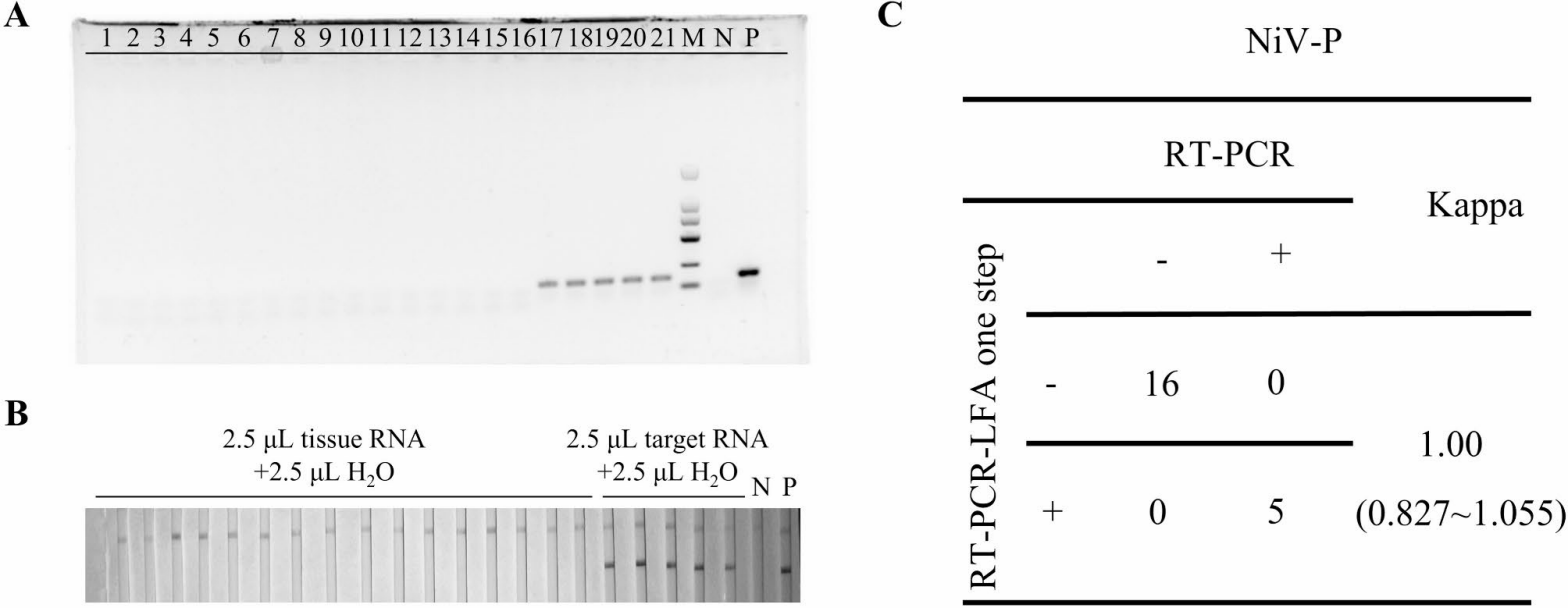
Coincidence rate of simulated clinical sample assay. **A**, **B**: The results of simulated clinical sample detection targeting NiV P gene with RT-PCR and POC-NAD; **C**. Coincidence rate of conventional RT-PCR and POC-NAD with Kappa analysis. 1–16: we use the mixture of clinical sample RNA in 2.5µL and ddH2O in 2.5µL; 17–21: we use the mixture of clinical sample RNA in 2.5µL and the P and G gene RNA fragment in 1.25µL (6370 copies/µL); The total volume of the sample is 5µL; N: Negative control, template is H_2_O. P: positive control, template is P gene RNA fragment.

**Fig. 6:**
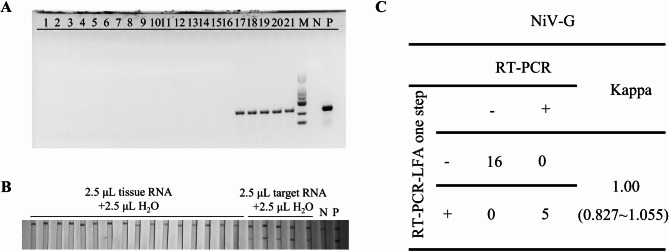
Coincidence rate of simulated clinical sample assay. **A**, **B**: The results of simulated clinical sample detection targeting NiV G gene with RT-PCR and POC-NAD; **C**. Coincidence rate of conventional RT-PCR and POC-NAD with Kappa analysis. 1–16: we use the mixture of clinical sample RNA in 2.5µL and ddH2O in 2.5µL; 17–21: we use the mixture of clinical sample RNA in 2.5µL and the P and G gene RNA fragment in 1.25µL (6370 copies/µL); The total volume of the sample is 5 µL; N: Negative control, template is H_2_O; P: positive control, template is G gene RNA fragment.

Finally, we simulated 21 clinical samples that sixteen negative simulated clinical samples and five positive simulated clinical samples were successfully detected by dual-target detection method, which was consistent with the results of RT-PCR method (Figs. 5 and 6). And the results performed that this dual-target detection strategy demonstrated good stability and accuracy in this study.

## Discussion

As a zoonotic virus, NiV has repeatedly caused illness outbreaks in Asia over the past two decades^19^. The recent high-mortality of this disease in India highlight the urgency of virus detection and research^20,^^21^. Pigs, a primary source of human meat consumption, are important intermediate hosts for NiV, leading to increasingly significance of the viral detection in pigs^22^. However, the disease caused by NiV infection in pigs is mild and has no distinctive clinical symptom, complicating the early diagnosis and facilitating the viral spread unnoticedly^23^. Development of in-field diagnostic methods for NiV has become a focus to stop the epidemic and spread of the virus^24^.

Several in-field diagnosis for NiV has been successfully established. Ma et al. developed a reverse transcription-loop-mediated isothermal amplification (RT-LAMP) technique for NiV and enabled visual detection based calcein indicator by the naked eye^25^. Another team has developed an optimized one-pot assay by using recombinase polymerase amplification (RPA) coupled to CRISPR/Cas13a for the molecular detection of NiV. It can detect the results through fluorescence or visual lateral flow strips^19^. However, certain limitations prevent these new approaches applied in clinics. The specificity and accuracy of RT-LAMP usually can be reduced due to non-specific amplification and/or by non-specific interference^26^. It is expensive to complete the RNA virus detection based on RPA reaction^27^. Compared with Pollak’s evaluation of three rapid low-resource molecular tests for Nipah virus, our detection methods with higher sensitivity is much more stable^28^.

Therefore, we established a field nucleic acid test for NiV, based on a one-step RT-PCR reaction coupled with lateral flow immunoassay strips and portable PCR. This method is cost-effective (less than 0.3 dollar/rxn), compared to the high cost of 5 dollars for RT-qPCR^29^. The whole process from nucleic acid extraction to results determination takes about an hour, which is faster than current detection technologies such as qPCR and ELISA. Furthermore, the one-step reverse transcription and amplification process eliminates the risk of sample cross-contamination associated with two separate reactions. A potential limitation of this study is that we did not use alive Nipah virus for testing due to the deficiency of clinical samples. To overcome the limitation, we implemented a dual-target in-field primers design to ensure a broader applicability and enhanced accuracy. Therefore, the test should provide reliable results regardless of the NiV genotype.

## Conclusion

In this study, the developed point-of-care nucleic acid diagnostic (POC-NAD) assay demonstrated a lower limit of detection (LOD) of 199.1 RNA copies/rxn for Nipah virus (NiV). Validation studies confirmed 100% specificity for NiV detection against seven porcine viral pathogens (ASFV, PRRSV, PEDV, PDCoV, TGEV, PRoV, and OZK93 strain). Operational evaluation revealed per-test reagent costs of $0.28 ± 0.02 and field deployment capability. These attributes position the assay as a cost-effective adjunctive tool for longitudinal surveillance of NiV epidemiology in swine production systems and associated environmental reservoirs.

## Electronic supplementary material

Below is the link to the electronic supplementary material.

Supplementary Material 1

Supplementary Material 2

## Data Availability

The datasets used and/or analysed during the current study are available from the corresponding author on reasonable request.

## Abbreviations

NiV: Nipah Virus
BSL-4: biosafety laboratory
PCR: polymerase chain reaction
CDC: the Centers for Disease Control and Prevention
POC-NAD: point-of-care nucleic acid detection
ASFV: African swine fever virus
PRRSV: Porcine reproductive and respiratory syndrome virus
PEDV: Porcine epidemic diarrhea virus
PRoV: porcine rotavirus
OZK93: Porcine foot-and-mouth disease virus
PDCoV: Porcine delta coronavirus
TGEV: Transmissible gastroenteritis virus
LoD: limit of detection
NTC: no-template control
RT-LAMP: reverse transcription-loop-mediated isothermal amplification
