# Supplementary material for: A Stable and Dependable Visual Technique for On-Site Nipah Virus Nucleic Acids Detection
Source: Sci Rep. 2025 Feb 27;15:7037. doi: 10.1038/s41598-025-91593-w (PMC11868622; doi:10.1038/s41598-025-91593-w)
Supplement: Supplementary file 2 — Supplementary Material 2 [file 41598_2025_91593_MOESM2_ESM.pdf]

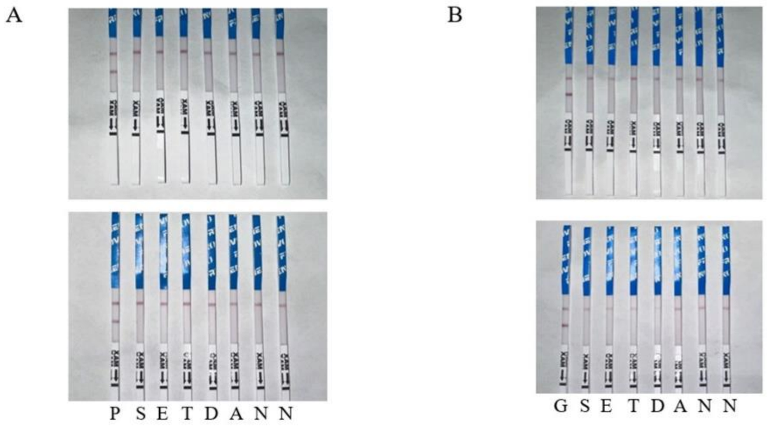

Supplement Figure 1. A. Results of the specificity of P-1 on lateral flow assay; B. Results of the specificity of P-2 on lateral flow assay; P and G: positive control; E: PEDV; D: PDCoV; S: PRRSV; T: TGEV; A: ASFV; N: Negative control.

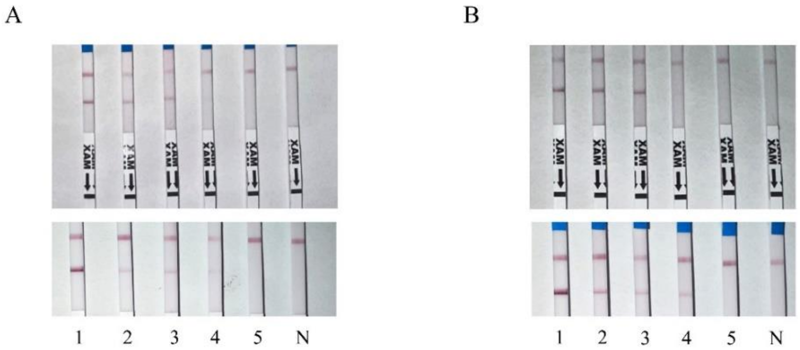

Supplement Figure 2. A. The visual result of P-1; B. The visual result of P-2; 1-5: 1592.5 copies/rxn, 796.25 copies/rxn, 398.1 copies/rxn, 199.1 copies/rxn, 99.55 copies/rxn; N represents the negative control.

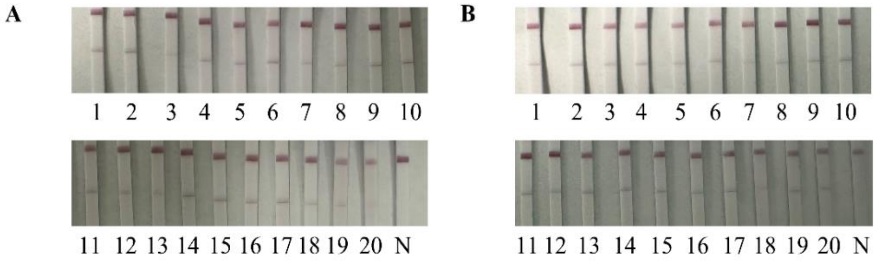

Supplement Figure 3. Test 20 replicates of NiV template. A. Results of P-1-X; B. Results of P-2-X.
